# C53 is a cross-kingdom conserved reticulophagy receptor that bridges the gap betweenselective autophagy and ribosome stalling at the endoplasmic reticulum

**DOI:** 10.1080/15548627.2020.1846304

**Published:** 2020-12-03

**Authors:** Madlen Stephani, Lorenzo Picchianti, Yasin Dagdas

**Affiliations:** a Gregor Mendel Institute (GMI), Austrian Academy of Sciences, Vienna BioCenter (VBC), Vienna, Austria; b Vienna BioCenter PhD Program, Doctoral School of the University at Vienna and Medical University of Vienna, Vienna, Austria; c Research Institute of Molecular Pathology (IMP), Vienna BioCenter (VBC), Vienna, Austria

**Keywords:** *Arabidopsis thaliana* CDK5RAP3, ER-phagy, ER-quality control, ribosome stalling, selective autophagy, selective autophagy receptor, ufmylation

## Abstract

Reticulophagy, the autophagic degradation of the endoplasmic reticulum, is crucial to maintain ER homeostasis during stress. Although several reticulophagy receptors have been discovered recently, most of them have been studied using nutrient starvation. How macroautophagy/autophagy cross-talks with other ER-quality control mechanisms is largely unknown. Using ATG8-based affinity proteomics in the model plant *Arabidopsis thaliana*, we identified AT5G06830/C53, a soluble protein that directly interacts with ATG8. Biochemical and biophysical characterization of C53-ATG8 interaction using both human (CDK5RAP3) and Arabidopsis proteins revealed that C53 binds ATG8 via shuffled Atg8-family interacting motifs (sAIMs) located at its intrinsically disordered region (IDR). C53 is recruited to phagophores, precursors to autophagosomes, during ER stress in an autophagy-dependent manner. Consistently, c53 mutants are highly sensitive to ER stress treatments. C53 senses ER stress by forming a tripartite receptor complex that involves UFL1, the E3 ligase that mediates ufmylation, and its ER-resident adaptor protein DDRGK1. C53 activity is regulated by another ubiquitin-like protein, UFM1, which is transferred from C53 to the ribosomes upon ribosome collision/stalling at the ER, thereby activating the C53 pathway to recycle stalled nascent chains. Altogether our findings suggest C53 forms an ancient quality control pathway that links ribosome-associated quality control with selective autophagy at the ER.

The endoplasmic reticulum is a highly dynamic network that is crucial for protein folding and maturation, lipid metabolism, and Ca^2+^ homeostasis. Accumulation of unfolded proteins in the ER triggers a series of quality control mechanisms such as the unfolded protein response/UPR and ER-associated degradation/ERAD. Recent studies have shown that reticulophagy, the recycling of ER compartments by selective autophagy, is also a major quality control mechanism that remodels the ER during starvation and stress.

**Figure 1.**
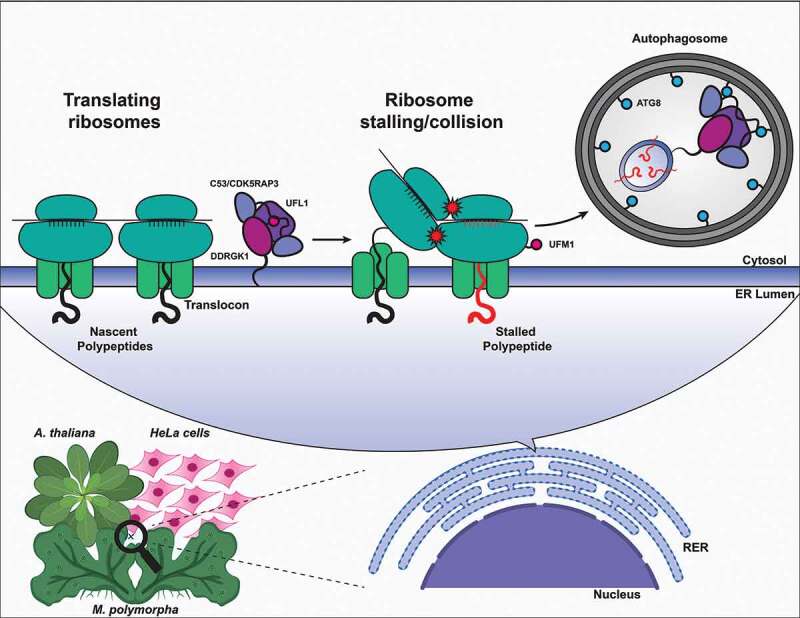
Current working model of the C53 receptor complex. Upon ribosome stalling/collisions, UFM1 is transferred from the C53 receptor complex (C53-UFL1-DDRGK1) to the tail of the ribosomal subunit RPL26. This exposes the sAIMs on C53 for ATG8/GABARAP binding and subsequent complex recruitment to the phagophore for degradation

Since the discovery of the first reticulophagy receptors in yeast and mammalian cells in 2015, several other receptors have been identified. In mammals, these include ER-resident proteins RETREG1/FAM134B, RTN3L, SEC62, CCPG1, TEX264, ATL3, and the soluble protein CALCOCO1. Similarly, in plants, ATI1, ATI2, ATI3, RTN1, and ATSEC62 have been linked to ER recycling. Whether these receptors work in concert and how reticulophagy is intertwined with other ER-quality control pathways still remain largely obscure. In addition, mechanisms of activation of these reticulophagy receptors, and how they are targeted to the regions of the ER that needs to be recycled need further investigation.

In our recent study [1], we identified C53 as a soluble reticulophagy receptor that is distinct from the known reticulophagy receptors in three main aspects:

1. Unlike most other reticulophagy receptors, C53 is highly conserved in multicellular eukaryotes. This protein binds ATG8 via distinct binding epitopes, located at the C53 IDR, that we named shuffled AIMs (sAIMs). Stoichiometry analysis revealed that C53 binds two ATG8 proteins with a low micromolar binding affinity. Binding more than one ATG8 could help in clustering the core autophagy machinery to facilitate the reticulophagy process. In addition, C53 also interacts with RB1CC1/FIP200 and Atg11 respectively, similar to other reticulophagy receptors such as CCPG1.
2. C53/CDK5RAP3 forms a receptor complex with UFL1, the E3 ligase that mediates ufmylation, and its adaptor protein, DDRGK1. UFL1/At3g46220 and DDRGK1/At4g27120 are co-delivered to the vacuole with C53 and are essential for C53-mediated autophagy. Consistently, c53, ufl1, ddrgk1, and the ufmylation pathway mutants are highly susceptible to ER stress treatments. Recently, other reticulophagy receptors such as Atg40 in yeast or RETREG1 in mammals have also been shown to form receptor complexes. Though, speculative at the moment, these receptor complexes could be involved in selective cargo recruitment and the crosstalk with other quality control pathways.
3. Unlike most other reticulophagy receptors, C53 is not activated by nutrient starvation. Upon carbon- or nitrogen-starvation conditions C53 remains diffuse in the cytosol. This protein is only activated upon ER stress induction such as ribosome stalling. For example, a model poly-lysine stalling substrate that is targeted to the ER (ER-K20) induces C53 puncta formation. Similarly, chemicals that inhibit translational elongation, thereby leading to ribosome collisions are potent inducers of C53-dependent autophagy. Upon activation, C53 mediates the degradation of stalled nascent chains that are otherwise toxic to the cell. Quantitative proteomics analyses indicate rather than structural ER proteins, translocon components or ribosomes, C53 cargo clientele include cell wall and extracellular proteins that fold and mature at the ER.

Our current working model is that ribosome stalling or collisions induces ufmylation of RPL26, a 60S ribosomal protein (Fig. 1). Once UFM1 is transferred to RPL26, sAIM residues, that are normally bound to UFM1, are exposed for ATG8 binding. ATG8 and RB1CC1/Atg11 interaction then initiate the C53-mediated autophagic recycling. However, how C53 can pull out stalled nascent chains from translocons is still unknown. Furthermore, how ufmylation machinery senses ER stress, and the crosstalk between ufmylation and C53-mediated reticulophagy need to be investigated further. Nevertheless, our findings reveal an ancient connection between ribosome-associated quality control and selective autophagy. As the ER is responsible for the synthesis of roughly 40% of the proteome, and C53 and the associated proteins are closely associated with various diseases, furthering our understanding of C53-mediated reticulophagy could have far reaching implications in agriculture and healthcare.
